# Alkene Difunctionalization Using Hypervalent Iodine Reagents: Progress and Developments in the Past Ten Years

**DOI:** 10.3390/molecules24142634

**Published:** 2019-07-19

**Authors:** Ji Hoon Lee, Sungwook Choi, Ki Bum Hong

**Affiliations:** 1 New Drug Development Center (NDDC), Daegu-Gyeongbuk Medical Innovation Foundation (DGMIF), 80 Cheombok-ro, Dong-gu, Daegu 701-310, Korea; 2 Department of New Drug Discovery and Development, Chungnam National University, Daejon 305-764, Korea

**Keywords:** hypervalent iodine, alkene difunctionalization, diamination, diacetoxylation, aminofunctionalization, oxyfunctionalization, dihalogenation

## Abstract

Hypervalent iodine reagents are of considerable relevance in organic chemistry as they can provide a complementary reaction strategy to the use of traditional transition metal chemistry. Over the past two decades, there have been an increasing number of applications including stoichiometric oxidation and catalytic asymmetric variations. This review outlines the main advances in the past 10 years in regard to alkene heterofunctionalization chemistry using achiral and chiral hypervalent iodine reagents and catalysts.

## 1. Introduction

Starting in the early 2000s, hypervalent iodine chemistry has undergone extensive development and a wide range of organic synthesis applications have been devised owing to their stability in air and to moisture, commercial availability, low toxicity, mild reaction conditions, and ease of handling. Most importantly, however, their complementary oxidizing ability compared to transition metals has been the main reason why they are increasingly being studied and used in synthetic organic chemistry. As an efficient oxidant, hypervalent iodine allows for a wide range of synthetic transformations, namely oxidation of alcohols, α-functionalization of carbonyl compounds, spirocyclization, and difunctionalization of alkenes. Both stoichiometric iodine chemistry and catalytic enantioselective variations were initially investigated in regard to α-functionalization and spirocyclization chemistry, although recent progress has resulted in a more diverse range of applications. For example, due to their oxidizing capacity, the combination with transition metals constitutes a new avenue for prominent carbon-heteroatom bond formation. Hypervalent iodine is a way to gain access to high oxidation state complexes with transition metals. Although they do have some limitations such as the generation of stoichiometric byproducts as well as their limited scope, hypervalent-mediated high-valent transition metal catalysis is nonetheless clearly an emerging area that holds ample promise. Aside from transition metal chemistry, hypervalent iodine itself has the aforementioned properties suitable for application in a diverse range of organic transformations. Consequently, over the past two decades, there have been several general reviews [1,2,3,4,5,6,7,8,9,10], books and book chapters [11,12,13], and specialized reviews [14,15,16,17,18,19,20,21,22,23,24,25,26,27,28,29] in this regard. In this review, we hence focus on progress and developments that have taken place in the past 10 years in the field of alkene difunctionalization such as diamination, aminofunctionalization, diacetoxylation, oxyfunctionalization, and dihalogenation reactions.

## 2. Alkene Diamination

**Scheme 1:**
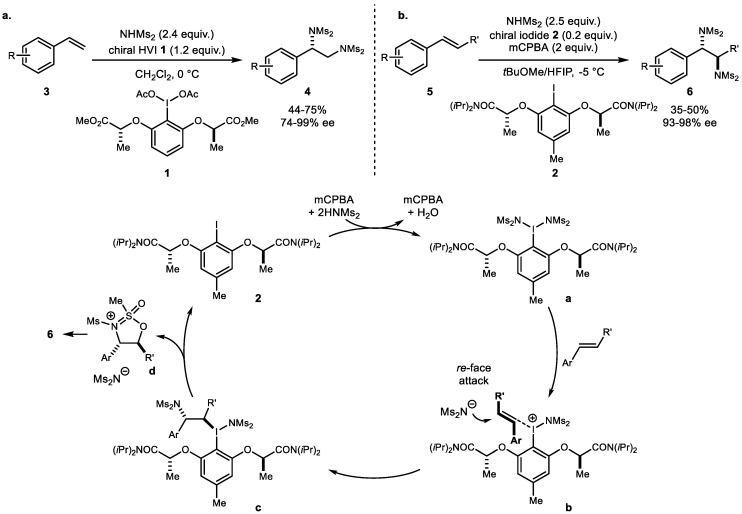
(**a**) Stoichiometric diamination with chiral hypervalent iodine and (**b**) catalytic diamination with chiral iodide.

The diamination reaction of alkenes has recently become a useful synthetic approach due to the potential to economically generate a diverse range of vicinal diamines of pharmaceuticals and natural products. In 2011, Muñiz and colleagues described the first enantioselective transfer of two nitrogen atoms using bismesylimide onto the prochiral face of styrene using a chiral iodine (III) reagent **1** (Scheme 1a) [30]. Direct intermolecular diamination of an unactivated alkene is uncommon, especially in the absence of a metal catalyst. In this context, stoichiometric chiral hypervalent iodine exclusively promoted overall oxidation of alkene up to 95% ee (enantiomeric excess). Subsequently, in 2017, Muñiz and colleagues developed the first catalytic asymmetric diamination using 4-methylaryl iodide **2** with two modified chiral lactamide side chains and 3-chloroperbenzoic acid as a terminal oxidant in an MTBE/HFIP (methyl tert-butyl ether/hexafluoroisopropanol) solvent combination (Scheme 1b) [31]. This protocol yielded a wide range of products with excellent enantioselectivity of up to 98% ee. This pioneering work demonstrated catalytic intermolecular enantioselective diamination for the first time and provided insights into the rational design of catalysts within the context of hypervalent catalyzed alkene functionalization.

**Scheme 2:**
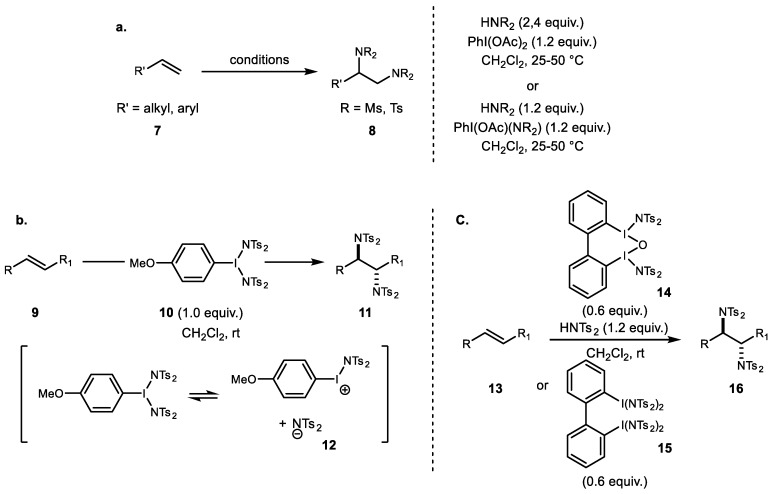
(**a**) Muñiz’s general combination of reagent for alkene diamination; (**b**) electronic effect on the hypervalent iodine; and (**c**) dinuclear iodine reagent for diamination.

Between 2012 and 2016, more general use of the achiral iodine reagent and bissulfonimide for diamination of alkene was also reported by Muñiz et al. (Scheme 2a) [32]. Over 60 examples comprising a diverse range of terminal and internal alkenes were converted to vicinal diamine under metal-free conditions. The synthesis and the isolation of these new hypervalent iodine PhI[N(SO_2_R)_2_]_2_ entities, as well as their reactivity, were also reported by Muñiz as was the X-ray structure of chiral bisimidoiodine [33]. Additionally, an investigation of electronic effects on the aryl substituent of the hypervalent iodine reagent revealed that the reaction was accelerated by electron donating groups due to stabilization of the cationic iodonium **12** (Scheme 2b) [34]. Another example of the new dinuclear iodine **15** involves intermolecular alkene diamination (Scheme 2c) [35]. This protocol provides a comparable reactivity and a new addition for amination reactions. Although initial attempts using the chiral binaphthyl reagent for enantioselective variation yielded low ee values, this newly designed hypervalent iodine motif nonetheless has significant potential for future development.

**Scheme 3:**
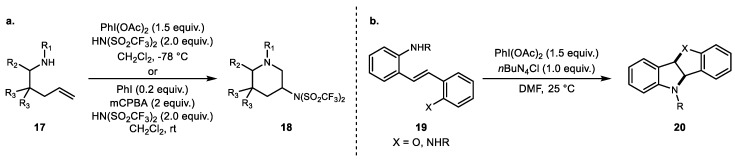
(**a**) Blakey’s alkene diamination protocol and (**b**) Chang’s intramolecular diamination.

In 2012, Blakey and coworkers developed both stoichiometric and catalytic versions of intramolecular alkene diamination for stereoselective 6-*endo*-oxidative cyclization (Scheme 3a) [36]. The same reaction condition reported by Muñiz and colleagues yielded stereoselective 6-*endo* 3-aminopiperidine derivatives when the substrate had a geminal alkyl substitution at the 4-position. Additionally, the homoallylic substituents in the pentene side chain improved the regioselectivity and resulted in good diastereoselectivity. The same year, Chang and colleagues demonstrated not only an elegant diamination reaction but also inorganic additive effects and functional group effects on the nitrogen atom [37]. Aside from Lewis acids and other promoters, halide additives also enhanced the overall efficiency and reactivity of the hypervalent iodine chemistry. These examples demonstrate the potential to achieve alkene heterofunctionalization using only hypervalent iodine compared to previously reported Pd(II)/Pd(IV)-based chemistry using the same substrates [38].

**Scheme 4:**
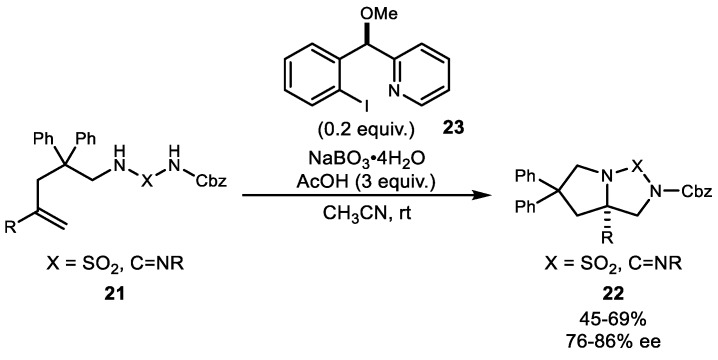
Wirth’s first catalytic intramolecular diamination.

In 2014, Wirth et al. demonstrated the first catalytic intramolecular diamination using various tethered homoallylic guanidine and diaminosulfone derivatives to generate bicyclic molecules (Scheme 4) [39]. This newly designed hypervalent iodine reagent **23** with a pyridine affixed to a chiral benzylic center can allow efficient coordination of the pyridine nitrogen to the iodine center and can yield cyclized adduct up to 94% ee. Subsequent reduction of both the X (SO_2_ and C=NR) and the Cbz group yielded 1,2-diamine at 86% ee. While only diphenyl-substituted substrates led to the desired products in good yields and with high enantioselectivities, the newly introduced hypervalent iodine ligand **23** can provide new enantioselective variation compared to lactate-based iodine catalysts.

**Scheme 5:**
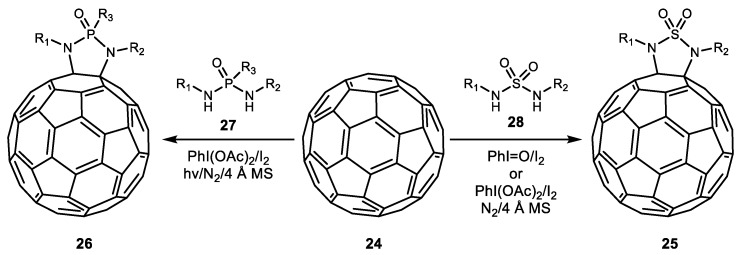
Miao’s C_60_ diamination.

The same year, Miao et al. reported an intermolecular variation of oxidative diamination of olefins with sulfamide **28** and phosphoryl diamide **27** for the synthesis of C_60_-fused cyclic sulfamide **25** and phosphoryl diamide **26** (Scheme 5) [40]. Depending on the combination of the iodine source, molecular iodine, and the nitrogen source, either a selective diamination or an aziridination reaction was achieved. Notably, phosphoryl diamide was used as a diamine source for the first time.

**Scheme 6:**
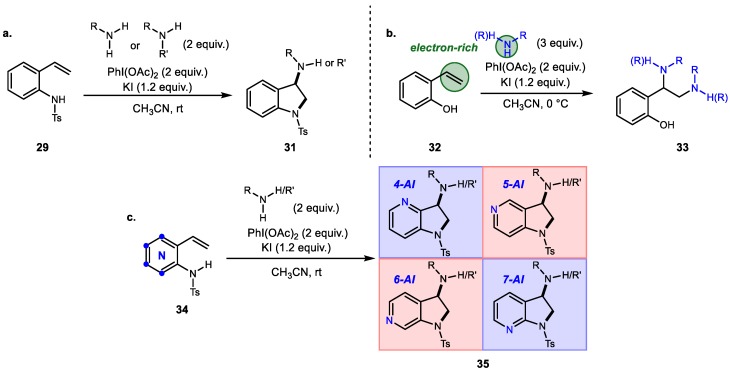
(**a**) Alkene diamination—indoline synthesis; (**b**) intermolecular diamination; and (**c**) azaindoline synthesis.

Unlike other diamination protocols that used an electron-deficient nitrogen source, Johnston and Hong developed a series of alkene diaminations using Brønsted-base, electron-rich amines (Scheme 6) [41,42,43]. A combination of stoichiometric hypervalent iodine and inorganic additives can promote inter/intramolecular C-N bond formation without amine preactivation or protection. This protocol can be used to generate 3-aminoindolines and 1,2-diamine derivatives, and it provides a gateway to access all four 3-aminoazaindoline derivatives.

## 3. Alkene Aminofunctionalization

**Scheme 7:**
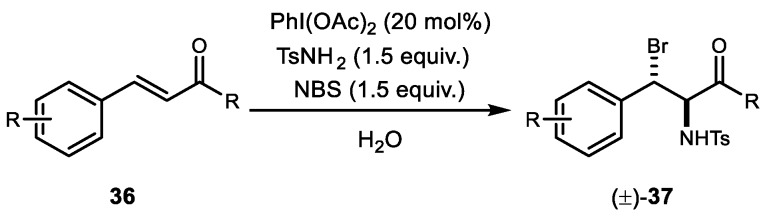
The first PhI(OAc)_2_ catalyzed intermolecular aminofunctionalization.

The first PhI(OAc)_2_-catalyzed intermolecular amino-bromination of alkene in water with a TsNH_2_ and NBS combination was reported by Wang et al. in 2009 (Scheme 7) [44]. A diverse range of electron-deficient olefins such as α,β-unsaturated ketones, cinnamates, and cinnamides were tolerated under these reaction conditions. Additionally, this reaction resulted in an unusual rate of acceleration and regioselectivity for styrenes compared to the reaction in organic solvents.

**Scheme 8:**
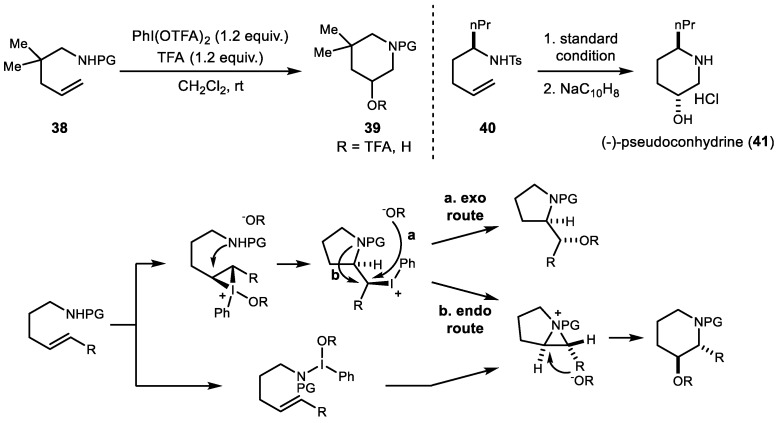
FIFA-mediated (Bis(trifluoroacetoxy)iodo)benzene) endo-selective aminooxygenation.

In 2010, Michael et al. demonstrated the first example of a highly endo-selective aminooxygenation (Scheme 8) [45]. Even though the stereochemical outcome depended on the substrates, the piperidine product favored anti-addition in endo-cyclization. This methodology was successfully applied to the synthesis of (−)-pseudoconhydrine **41** in a total of 6 steps.

**Scheme 9:**
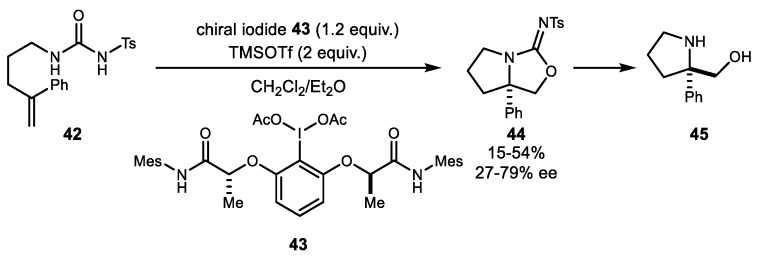
The first example of stereoselective aminooxygenation.

The first example of stereoselective aminooxygenation using a chiral hypervalent iodine reagent was reported by Wirth and colleagues in 2012 (Scheme 9) [46]. A lactate-based amide attached chirally to hypervalent iodine **43** and TMSOTf (Trimethylsilyl trifluoromethanesulfonate) combination at a low temperature provided the highest selectivity for isourea adduct **44** formation. This isourea can readily be converted to the α-substituted proline derivative **45,** which has not been synthesized with high enantiopurity.

**Scheme 10:**
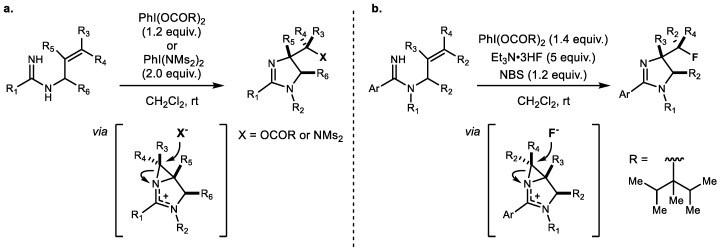
(**a**) Diastereoselective alkene aminooxygenation and (**b**) anti-selective aminofluorination.

In 2014, Chiba et al. reported a series of diastereoselective aminooxygenations using either PhI(OAc)_2_ or PhI(OTFA)_2_ and diamination using defined Muñiz’s bisimido iodine reagent **47** (Scheme 10a) [47]. Compared to previous metal-catalyzed reactions [48,49], this strategy extended the scope to substituted internal alkenes bearing *N*-allylamidine **46**. Subsequently, in 2016, the same group reported anti-selective aminofluorination using bulkier carboxylate (2-isopropyl-2,3-dimethylbutanoate)-attached iodobenzene **50** to minimize oxygenation of the product and excess Et_3_N·3HF combination (Scheme 10b) [50]. Furthermore, these 4-fluoro-2-alkyl imidazolines **51** could yield functionalized 3-fluoropropane-1,2-diamine derivatives after a reductive ring-opening step. Both procedures involve a stepwise sequence comprising syn-aziridination and nucleophilic ring opening of an aziridinium intermediate, with various nucleophile sources such as oxygen, nitrogen, and fluorine sources.

**Scheme 11:**
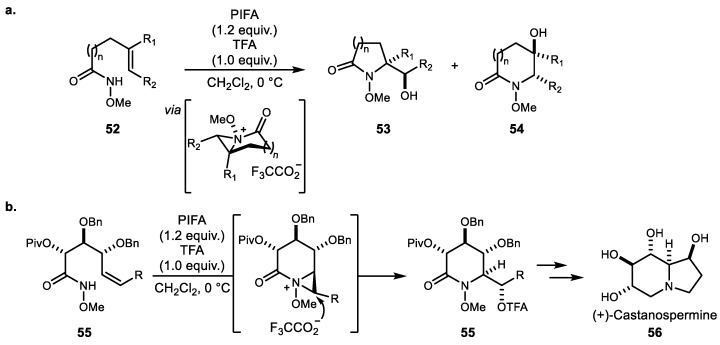
(**a**) Intramolecular oxamidation and (**b**) application to the synthesis of natural product.

In 2010, Wardrop et al. demonstrated PIFA/TFA-mediated (phenyliodine bis(trifluoroacetate)/trifluoroacetic acid) intramolecular aminooxygenation using nitrenium generated from *O*-alkyl hydroxamate **52** to generate bicyclic five- to eight-membered lactams (Scheme 11a) [51]. With this protocol, they noted further rate acceleration and overall efficiency in the presence of 1 equivalent of trifluoroacetic acid. Additionally, this nitrenium-mediated aminooxygenation was successfully applied to the synthesis of a series of polyhydroxylated indolizidine alkaloids **56** (Scheme 11b) [52,53]. The same protocol was recently used to synthesize madangamine D and other morphan-based natural products [54].

**Scheme 12:**
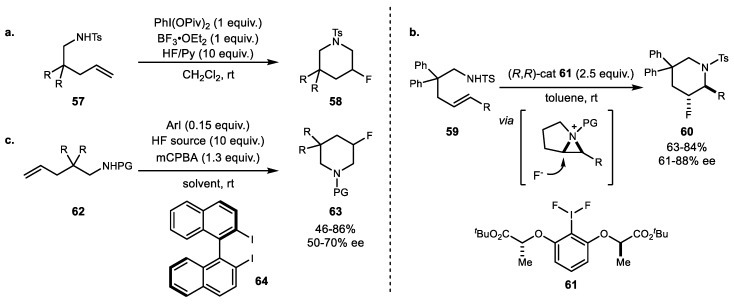
(**a**) First aminofluorination using stoichiometric iodine reagent; (**b**) asymmetric aminofluorination; and (**c**) catalytic aminofluorination.

A series of metal-free intramolecular aminofluorination of alkenes was reported in 2012. The first example of stoichiometric hypervalent iodine reagent variation was reported by Meng and Li in the presence of HF·Py and BF_3_·OEt_2_ as a promoter (Scheme 12a) (Scheme 12a) [55]. The reaction resulted in endo-selective formation of 3-fluoropiperidine derivatives **58** in one step under mild conditions. Subsequently, an asymmetric version of this protocol was reported by Nevado and coworkers using tert-butyl lactate-based iododifluoride reagent **61**. This reaction also yielded highly selective 6-endo-cyclization product **60** (Scheme 12b) [56]. Another catalytic example was developed by Kita and Shibata using axially chiral bisiodine reagent **64** in the presence of mCPBA (3-Chloroperoxybenzoic acid, meta-Chloroperbenzoic acid) (Scheme 12c) [57]. However, this protocol exhibited a moderate degree of enantioselectivity compared to lactate-based catalysts.

**Scheme 13:**
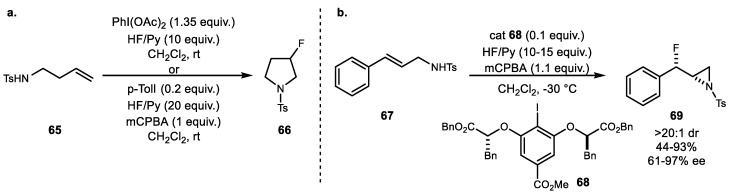
(**a**) Aminofluorination of homoallylamine and (**b**) catalytic enantioselective aminofluorination.

Aside from six-membered ring formation, Zhang and coworkers disclosed the first metal-free intramolecular aminofluorination of homoallylamine **65** for the synthesis of 3-fluoropyrrolidine **66** with PhIO oxidant and BF_3_·OEt_2_ as the fluorine source [58]. A more practical method was reported by Kitamura and coworkers [59]. They developed both stoichiometric methods using a PIDA/HF (phenyliodine(III) diacetate/hydrogen fluoride) combination and a catalytic method using the *p*-iodotoluene/mCPBA combination for the synthesis of 3-fluoropyrrolidines **66** (Scheme 13a). Recently, Jacobsen et al. reported the synthesis of syn-β-fluoroaziridine via intramolecular aminofluorination of allylic amine **67** using dibenzyl lactate-based chiral hypervalent iodine **68**. Both a high yield and excellent enantioselectivity were observed, and this fluoroaziridine **69** was treated with a diverse range of nucleophiles to generate unique chiral building blocks (Scheme 13b) [60].

**Scheme 14:**
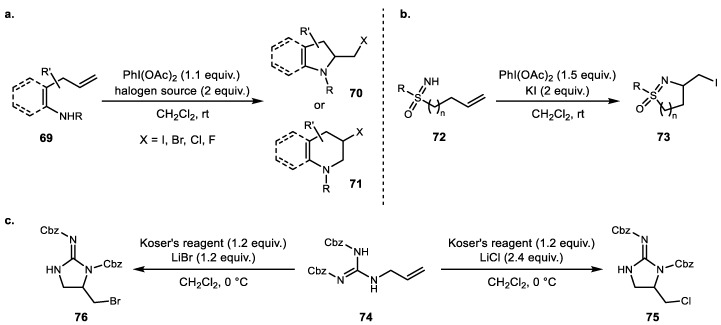
(**a**) Haloamination (F, Cl, Br, and I) of alkene; (**b**) iodoamination; and (**c**) chloro(bromo)amination.

Aside from fluorine addition to alkene, other halogens have also been added using hypervalent iodine chemistry. In 2014, Li and Liu demonstrated PIDA-mediated regioselective aminohalogenation using all four halogen sources [61]. A diverse range of carbo- and heterocycles such as pyrrolidine, piperidine, indoline, and dihydrofuran were synthesized after forming new C–N and C–X bonds. Another interesting iodocyclization of sulfoximine was reported by Bolm in 2016 [62]. The use of PIDA/KI combination, a diverse range of sulfoximines **72** resulted in five- and six-membered isothiazole and thiazine derivatives. Dodd and Carious also developed hypervalent iodine-mediated umpolung halocyclization using halide salts [63]. Regioselective chloro- and bromocyclization were achieved using Koser’s reagent and the corresponding halide sources (Scheme 14).

**Scheme 15:**
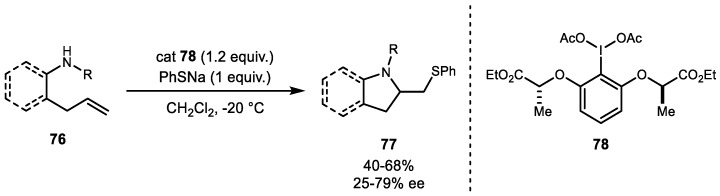
Wirth’s enantioselective thioamination.

The final heteroatom example of aminofunctionalization of alkene is the thioamination reaction that was reported by Wirth and colleagues in 2016 [64]. Using PIFA and a sulfur nucleophile, direct thioamination resulted in various pyrrolidine and indoline ring systems **77**. Additionally, lactate-based chiral hypervalent iodine **78** resulted in stereoselective formation of 1,2-aminothiols with up to 79% ee (Scheme 15).

**Scheme 16:**
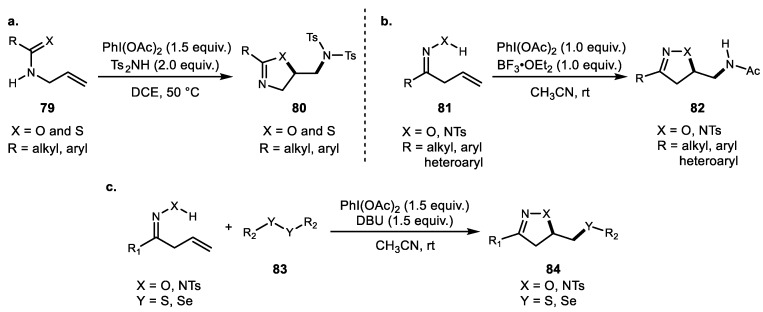
(**a**) Oxazoline and thiazoline synthesis; (**b**) Ritter-type amidation; and (**c**) sulfeno- and selenofunctionalization.

Other interesting heterocycle syntheses using alkene difunctionalization have recently been demonstrated. Using Muñiz’s protocol, Hong and coworkers reported oxazoline and thiazoline synthesis via alkene heterofunctionalization (Scheme 16a) [65]. Additionally, their systematic evaluation of amine functional groups provided evidence that the (benzene)sulfonyl group is the key moiety for the amination reaction. Another interesting Ritter-type amidation reaction was also reported by Hong and coworkers (Scheme 16b) [66]. The PIDA/BF_3_·OEt_2_ combination in CH_3_CN provided the unusual Ritter amidation adduct **82** after acetonitrile addition. Other heteroatoms such as sulfur and selenium were also incorporated with alkene in the presence of hypervalent iodine. Cai et al. developed efficient sulfeno- and selenofunctionalization of alkene to generate pyrazoline and isoxazoline in the presence of base (Scheme 16c) [67].

## 4. Alkene Diacetoxylation

**Scheme 17:**
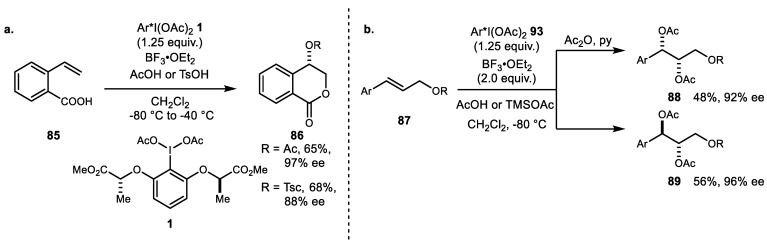
(**a**) Enantioseleictive oxylactonization and (**b**) enantioselective Prévost and Woodward reaction.

In terms of the dioxygenation reactions of alkene, recent advances have been mainly in regard to the reaction mechanism and synthetic applications with natural products, as reported by Fujita and coworkers in 2010. Initial screening of chiral lactate-based hypervalent iodine reagent **1** was reported in 2010 [68], and it resulted in both high enantioselectivities and endo-selective cyclization of 2-ethenylbezoic acid **85** into δ-lactone **86** (Scheme 17a). This protocol has been successfully applied to the enantioselective Prévost and Woodward reaction (Scheme 17b) [69]. The observed enantioselectivity switchover depended on the nucleophile and reaction temperature. The syn*-*product with retention of product was favored in the presence of water at low temperatures, while the anti-product was generated preferentially in the presence of acetic acid (or acetate).

**Scheme 18:**
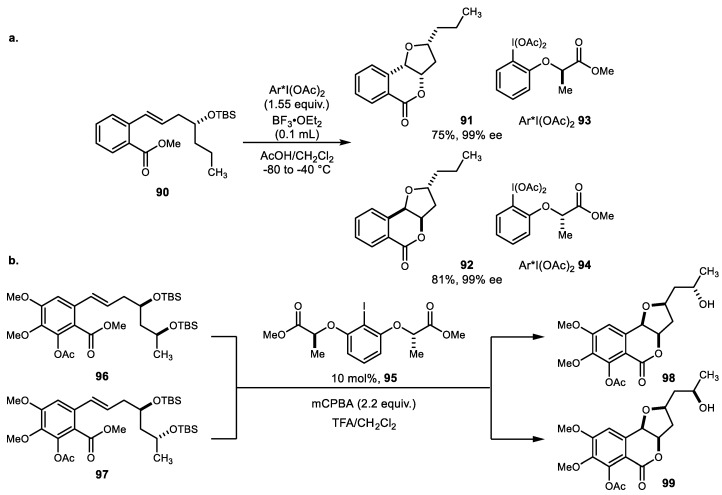
(**a**) Synthesis of 4-hydroxymellein, monocerin and (**b**) total synthesis of (12*S*)-12-hydroxymonocerin and (12*R*)-12-hydroxymonocerin.

The same group also reported a series of 4-oxyisochroman-1-one polyketide natural products. A series of substrates was evaluated for selectivity. In case of silyloxy substrate **90**, diastereoface selectivity was controlled by the chirality of the hypervalent iodine (Scheme 18a) [70]. The (*R*)-reagent **93** favored the *Si*-face attack to produce cyclized adduct **91**, while the (*S*)-reagent **94** favored the *Re*-face. Catalytic variation of same reaction using catalyst **95** was subsequently developed using mCPBA as a stoichiometric oxidant and TFA as an activator. Both stoichiometric use of chiral hypervalent iodine and a catalytic version were successfully applied for the first total synthesis of (12*S*)-12-hydroxymonocerin and (12*R*)-12-hydroxymonocerin (Scheme 18b) [71]. This overall selectivity based on the lactate-side chain on the chiral iodoarene and in the catalytic variation study was also disclosed [72].

**Scheme 19:**
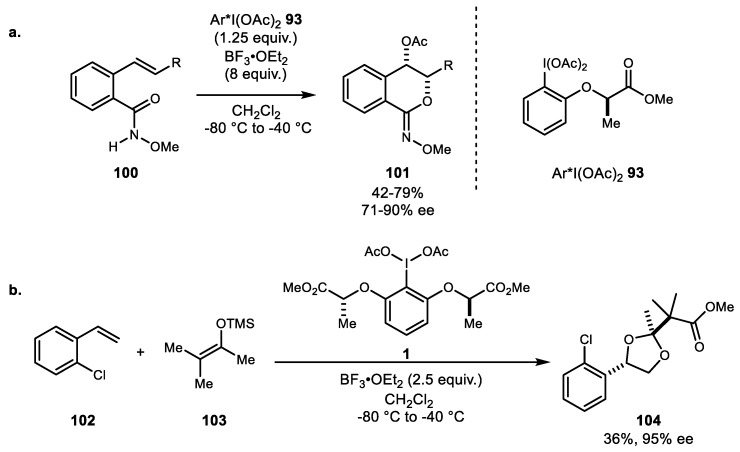
(**a**)Total synthesis of 4-hydroxymellein and 4-hydroxy-6-methoxymellein and (**b**) substituted 1,3-diozolane synthesis.

The development of a sequential oxidation reaction of alkenylbenzamide was applied for the synthesis of 4-hydroxymellein and related natural products (Scheme 19a) [73]. Oxidative cyclization of alkenylbenzamide **100** using chiral hypervalent iodine **93** yielded isochroman-1-imine, and palladium-catalyzed oxidative acetoxylation was subsequently shown to produce 4-hydroxymellein. Another interesting heterocycle family was obtained by alkene difunctionalization using a hypervalent iodine reagent. Using the external bidentate nucleophile **103**, the intermediate dioxolanyl cation converted substituted dioxolane as a single diastereomer (Scheme 19b) [74]. Various carboxylic acids and other carbon nucleophiles were tested, and enantioselective variation was also successfully demonstrated.

**Scheme 20:**
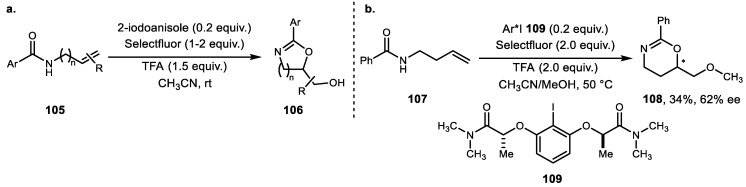
(**a**) *N*-Alkenylamide oxidative cyclization and (**b**) enantioselective variation.

Moran and colleagues continued to develop iodoarene-mediated and catalyzed oxidative cyclization reactions. In terms of alkene difunctionalization reactions, in 2015, Moran and coworkers reported cyclization of *N*-alkenylamide **105** to generate a diverse range of 5- to 7-membered ring systems **106** using catalytic amounts iodoarene and Selectfluor as the co-oxidant (Scheme 20a) [75]. Harned et al. had previously reported a similar reaction using a stoichiometric amount of the PIDA/ BF_3_·OEt_2_ combination [76]. However, their method only resulted in 5-membered ring systems with terminal alkene substrates. In Moran’s case, in addition to terminal alkenes, disubstituted alkenes could also readily be oxidized to the corresponding oxazoline derivatives **106**. Additionally, a preliminary screen of the chiral iodoarene **109** catalyzed-reaction yielded γ-butyrolactone **108** with moderate enantioselectivity (Scheme 20b).

**Scheme 21:**
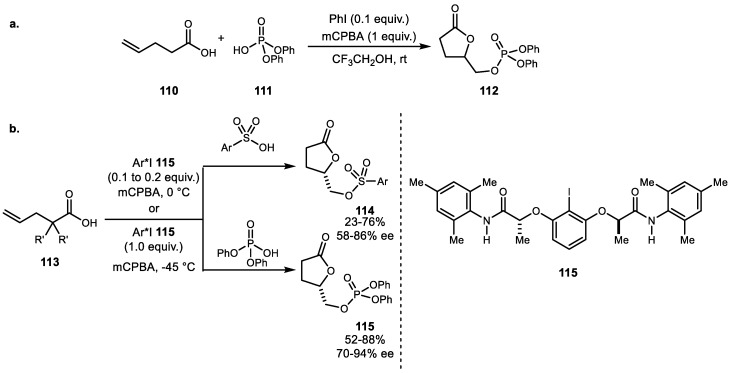
(**a**) First catalytic phosphoryloxylactonization and (**b**) enantioselective sufonyloxylactonizaiton and phosphoryloxylactonization.

The first example of phosphoryloxylactonization was reported by Koser et al. in 1988 [77]. However, the first catalytic variation was reported by Zhou et al. in 2010 (Scheme 21a) [78]. A combination of a catalytic amount of PhI and mCPBA promoted alkenoic acid **110** cyclization with phosphate to yield lactone **112**. Recently, Masson et al. developed the first enantioselective sufonyloxy- and phosphoryloxy-oxylactonization reaction using a catalytic lactamide-based chiral iodine and mCPBA combination (Scheme 21b) [79]. A diverse range of substrates **113** were subjected to either sulfonic acid or phosphoric acid nucleophile, resulting in cyclized adducts **114** and **115** with moderate to good enantioselectivity.

**Scheme 22:**
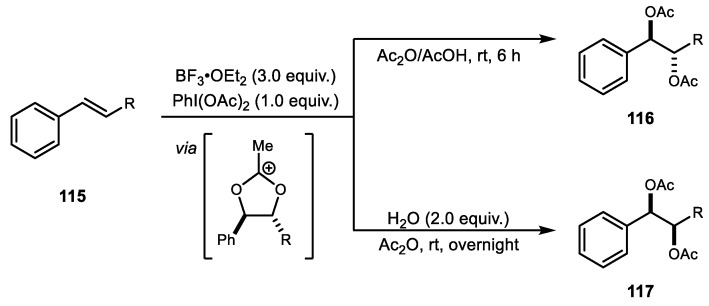
Diastereoselective diacetoxylation.

Another practical diastereoselective diacetoxylation of alkenes using PhI(OAc)_2_ was demonstrated by Li and Meng (Scheme 22) [80]. Using a PIDA/BF_3_·OEt_2_ combination, anti-selective diacetoxylation was achieved in Ac_2_O/AcOH. By contrast, syn-selective diacetoxylation was obtained in the presence of water in the same system. Various internal and terminal alkenes could be oxidized to generate the diacetate adducts. Notably, electron deficient alkenes were also tolerated under these reaction conditions and the efficiency of the reaction was maintained in a multi-gram scale reaction. Based on the Woodward–Prevost reaction mechanism [69], the BF3·OEt2/PIDA system activated alkene and formed the acetoxonium intermediate A. The syn-product was obtained after hydrolysis of acetoxonium A in the presence of water, while the anti-product was produced by ring opening of acetoxonium A. The same group subsequently reported syn-selective diacetoxylation of various alkenes using catalytic amounts of 4-MeC_6_H_4_I and BF_3_·OEt_2_/mCPBA in combination [81].

**Scheme 23:**
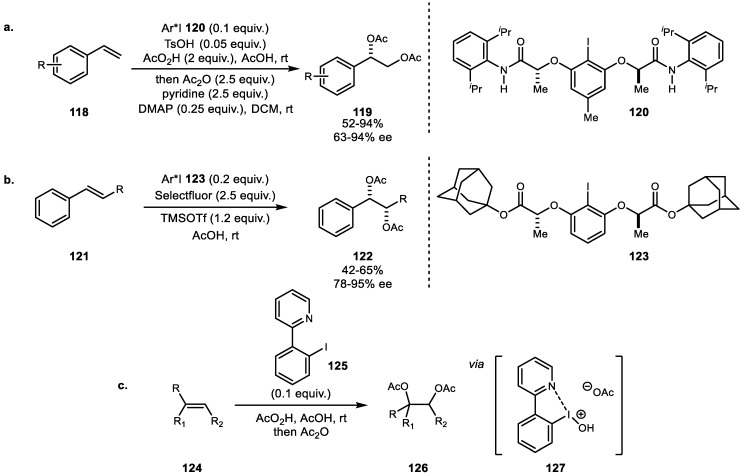
(**a**) First catalytic enantioselective diacetoxylation; (**b**) newly designed adamantly based chiral iodine; and (**c**) coordinating Lewis base-assisted iodine catalyst.

In 2016, Muñiz and colleagues developed a series of intermolecular diacetoxylation reactions. Muñiz and Ishihara elucidated the defined structural information of lactic amide-based chiral hypervalent iodine catalyst. Even though spatial information had been proposed previously [61], this work elucidated the structural information of the chiral iodine’s supramolecular helical chirality from the intramolecular hydrogen bonding network using X-ray analysis. Additionally, this work provided the first catalytic enantioselective diacetoxylation reaction (Scheme 23a) [82]. Along with the success of this new catalyst system, ongoing development of lactate-based chiral iodine chemistry was also developed by Muñiz et al. [83]. The newly synthesized adamantly substituted catalyst provided efficient diacetoxylation product up to 80% ee using Selectfluor as a promoter (Scheme 23b). Finally, a newly designed pyridine-coordinated iodine system was introduced by the same group (Scheme 23c) [84]. Pyridine moieties are commonly used for stabilization of the electrophilic iodine center. In this report, the catalyst structure revealed the coordination of the pyridine group to the iodine via a Lewis base-assisted activation.

**Scheme 24:**
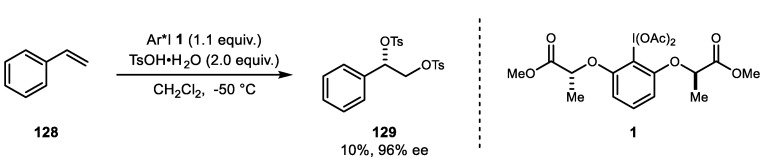
Enantioselective dioxytosylation.

The most recent example of dioxytosylation of styrene was reported by Fujita et al. in 2018 (Scheme 24) [85]. The stoichiometric use of lactate-based chiral iodine reagent **1** yielded the desired dioxytosylated adducts **129** up to 96% ee in the presence of TsOH at low temperatures. However, the substrate scope and reaction optimization are needed for future development.

## 5. Alkene Oxyfunctionalization

**Scheme 25:**
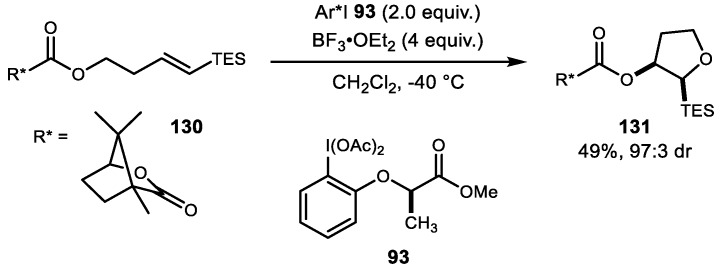
Fujita’s cycloetherification.

In 2009, Fujita et al. reported improved stereoselective cycloetherification using chiral auxiliary **130** and lactate-based chiral iodine reagent **93** (Scheme 25) [86]. The double asymmetric induction provided higher enantioselectivity compared to iodosylbenzene as a reagent.

**Scheme 26:**
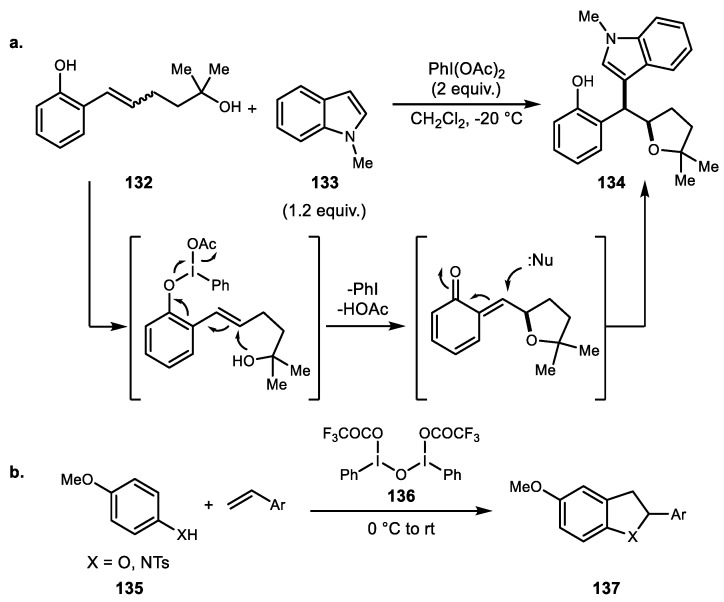
(**a**) Vinylphenol difunctionalization via quinone methide and (**b**) oxidative coupling via quinonium intermediate.

A series of metal-free oxidative alkene heterofunctionalizations via an *ortho*-quinone methide intermediate leading to various heterocycles has been reported. In 2012, Ding et al. demonstrated a PIDA-mediated vinylphenol difunctionalization (Scheme 26a) [87]. Compared to previously reported metal-catalyzed reactions [88,89], this protocol resulted in good yields and excellent diastereoselectivity. Additionally, the carbon nucleophiles were not limited to indole derivatives, as other heterocycles were also investigated. Kita et al. also used highly reactive hypervalent iodine dimer for the oxidative couplings of phenols with styrenes to generate indoline adducts (Scheme 26b) [90]. Use of a PIPA dimer generally enhanced the phenolic oxidation process.

**Scheme 27:**
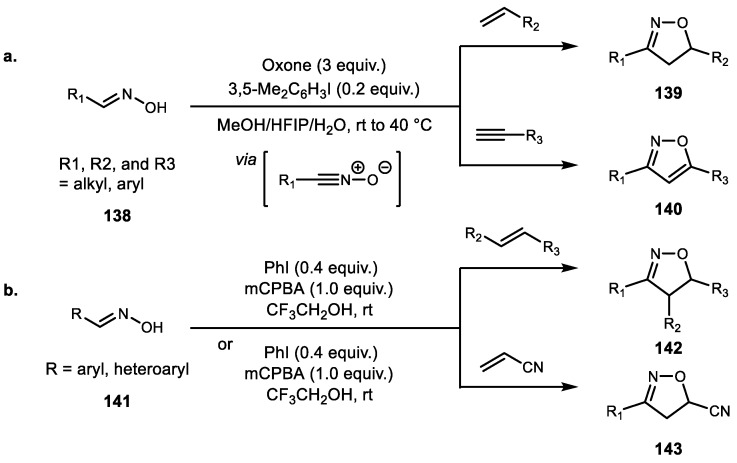
(**a**) Zhdankin’s cycloaddition and (**b**) Yan’s cycloaddition.

Several catalytic methods to generate an isoxazoline core have been reported. Zhdankin et al. demonstrated the first hypervalent iodine-catalyzed oxime cycloaddition with alkene using catalytic amounts of iodoarene and oxone as a terminal oxidant in aqueous HFIP. The overall efficiency was comparable to previously reported stoichiometric procedures, and alkynes are also applied to the synthesis of isoxazole (Scheme 27a) [91]. Similarly, Yan et al. reported a number of oxime cycloadditions with alkene using a substoichiometric amount of PhI and stoichiometric mCPBA oxidant. A diverse range of the corresponding isooxazolines were synthesized in moderate to good yields (Scheme 27b) [92]. Another cycloaddition using the hypervalent iodine-mediated process was reported by Prakash et al. for pyrazolyl isoxazoline derivatives [93].

**Scheme 28:**
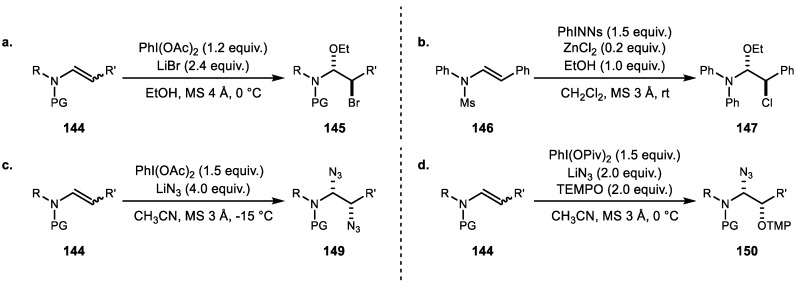
(**a**) Ethoxybromination; (**b**) ethoxychlorination; (**c**) diazidation; and (**d**) oxiazidation.

Dodd and Cariou have continued to report hypervalent iodine-mediated oxyhalogenation reactions using the umpolung strategy. The combination of PIDA and inorganic bromide in EtOH (Ethyl alcohol) resulted in ethoxybromination reaction of enamides. The iodine promoted umpolung reactions of halide salt to generate electrophilic halide that can react with various double bonds (Scheme 28a) [94]. In case of the ethoxychlorination reaction, ZnCl_2_ was used as the halide source. Preliminary mechanistic studies have revealed that the reaction proceeds via in situ-generated chlorinated hypervalent iodine species (Scheme 28b) [95]. As an extension of this protocol, a pseudohalide source and TEMPO ((2,2,6,6-Tetramethylpiperidin-1-yl)oxyl) combination resulted in the oxyazidation reaction (Scheme 28c,d) [96].

**Scheme 29:**
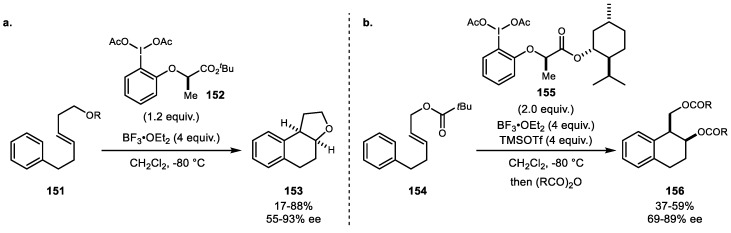
(**a**) Enantioselective oxyarylation and (**b**) acyloxy C–C bond formation.

In 2016, Fujita and colleagues added a new version of chiral hypervalent iodine-mediated enantioselective oxidative C–C bond formation. The silyloxy-attached internal alkene **151** effectively yielded oxyarylation adduct in the presence of lactate-based chiral iodine **152** and BF_3_·OEt_2_ as a promoter (Scheme 29a) [97]. Another example was reported by Fujita et al. in 2017 (Scheme 29b) [98]. An acyloxy group was introduced for the alkene oxidative oxyarylation reaction. Neighboring nucleophilic acyloxy groups participated effectively in generating the desired transformation to yield the naphthalene structure that is found in serrulatane diterpene natural products.

**Scheme 30:**
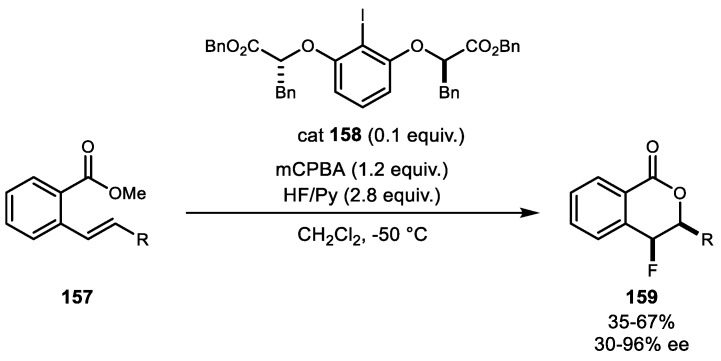
Catalytic enantioselective fluorolactonization.

Recently, Jacobsen et al. reported an enantioselective, catalytic fluorolactonization reaction using HF/Py as a nucleophilic fluorine source, mCPBA as the terminal oxidant, and lactate-based chiral iodine **158** (Scheme 30) [99]. A diverse range of terminal and internal styrenes with ortho carboxylic esters were converted to the enantioenriched 4-fluoroisochromanone scaffold **159**.

**Scheme 31:**
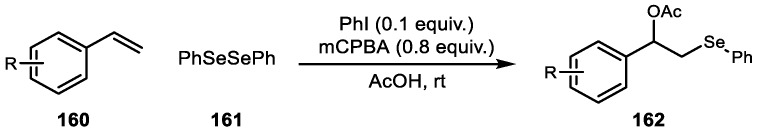
Catalytic oxyselenylation.

Lastly, Yan et al. reported an iodine-catalyzed acetoxyselenylation reaction via in situ generated electrophilic selenium species (Scheme 31) [100]. Although electrophilic selenium has been used for other hypervalent iodine reactions, the catalytic oxyselenylation has gained limited interest to date.

## 6. Alkene Dihalogenation

**Scheme 32:**
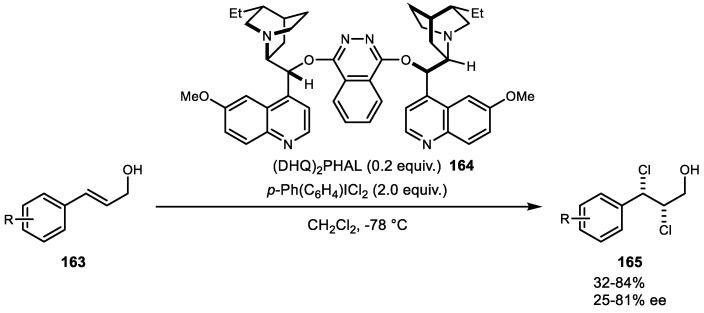
Nicolaou’s enantioselective dichlorination.

Alkene dihalogenation reactions using hypervalent iodine chemistry have recently attracted increasing attention. Seminal work in this regard was reported by Lupton et al. in 2009, but the overall process failed to achieve good selectivity [101]. In 2011, Nicolaou and coworkers developed an enantioselective alkene dichlorination reaction using cinchona alkaloid and *p*-Ph(C_6_H_4_)ICl_2_ as a chlorine source (Scheme 32) [102]. Although the substrates were limited to allyl alcohol with moderate enantioselection, this report provided insights regarding chlorenium ion formation by electrophilic iodine and a chiral catalyst.

**Scheme 33:**
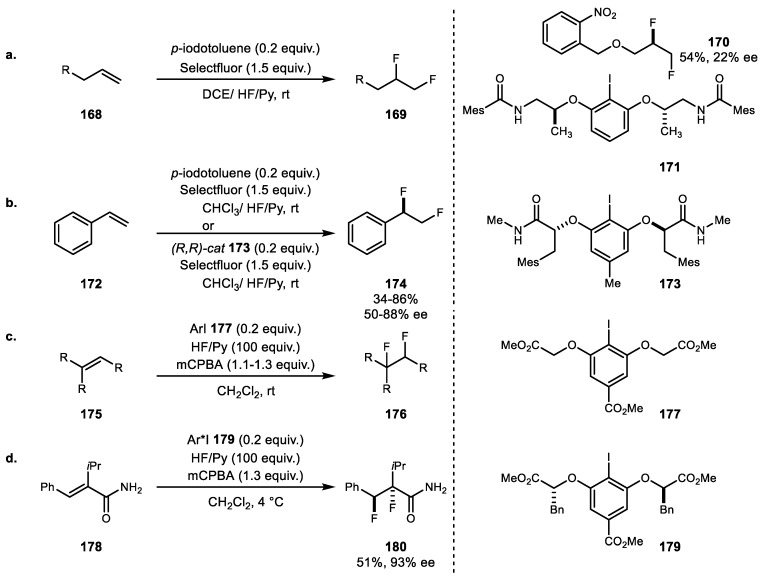
(**a**) Gilmour’s initial difluorination; (**b**) Gilmour’s enantioselective variation; (**c**) Jacobsen’s difluorination; and (**d**) Jacobsen’s enantioselective variation.

Although stoichiometric variation of alkene difluorination using *p*-iodotoluene difluoride was reported in 1998 by Hara and coworker [103], catalytic variation was recently reported by Gilmour et al. (Scheme 33a) [104]. Based on the combination of *p*-iodotoluene and Selectfluor, terminal alkene difluorination reaction was achieved using HF/Py as a halogen source. However, initial attempts at enantioselective variation using chiral iodine **171** only yielded 22% ee of **170**. The same group subsequently reported catalytic enantioselective difluorination using newly designed chiral iodine **173** [105]. This study revealed not only excellent new catalyst for enantioselective difluorination but also the correlation of ee values and the substrate electronic effect for determination of both the reaction course and the selectivity of the reaction pathway. Jacobsen et al. concurrently reported catalytic, diasteroselective vicinal difluorination using aryl iodide 174 and mCPBA oxidant (Scheme 33c) [106]. In addition to terminal alkenes, a diverse range of internal alkenes and electron-deficient alkenes were tolerated. Moreover, chiral iodine **177** yielded 93% ee of **176** adduct (Scheme 33d).

**Scheme 34:**
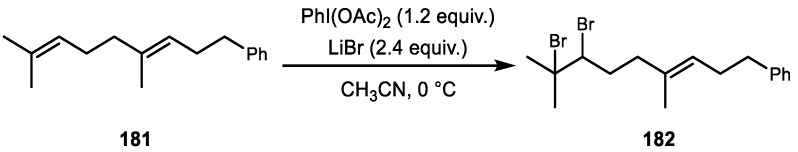
Cariou’s selective dibromination of polyprenoids.

Lastly, Cariou and coworkers recently demonstrated a chemodivergent, tunable, and selective method for bromofunctionalization of polyprenoids (Scheme 34) [107,108]. Depending on the combination of a hypervalent iodine source, inorganic salt, solvent, and temperature, the reaction produced selective dibromination, oxy-bromination, and bromocyclization reactions.

## 7. Conclusions

In this review, we provided an overview of the advances regarding hypervalent iodine-mediated and catalyzed alkene difunctionalization reactions in the past 10 years. Although we only focused on diamination, aminofunctionalization, diacetoxylation, oxyfunctionalization, and dihalogenation reactions, numerous advances and improvements have been observed regarding new reactions, reagent developments, and enantioselective transformations using chiral iodine reagents. While some of the aforementioned methodologies are needed to improve enantioselectivity and to diversify the range of the substrate scope, hypervalent iodine reagents and the catalysts themselves still exhibit complementary reactivity compared to transition metal-catalyzed organic transformations. Owing to their transition metal-like properties and environmentally benign nature, it is likely that more advances in this area of synthetic chemistry will be forthcoming in the near future.
